# Prognostic Gene Expression Signature for Age-Related Hearing Loss

**DOI:** 10.3389/fmed.2022.814851

**Published:** 2022-04-07

**Authors:** Lu Peng, Nianshen Li, Zhanrong Huang, Chunqin Qiu, Shihua Yin

**Affiliations:** Department of Otorhinolaryngology Head and Neck Surgery, The Second Affiliated Hospital of Guangxi Medical University, Nanning, China

**Keywords:** presbycusis, differentially expressed genes, immune infiltration, autophagy, diagnostic value

## Abstract

**Background:**

Our study aimed to determine the pathological mechanism of presbycusis at the molecular level, and determine potential biomarkers for the same.

**Methods:**

Differentially expressed genes (DEGs) for presbycusis were obtained by analyzing the microarray data sets (GSE6045 and GSE49543) downloaded from the Gene Expression Omnibus (GEO). Gene ontology (GO), Kyoto Encyclopedia of Genes and Genome (KEGG) pathway, and protein-protein interaction (PPI) network analyses, and Gene Set Enrichment Analysis (GSEA) were performed to analyze the biological functions, molecular pathways, autophagy-related molecular markers, and the immune microenvironment of the DEGs in presbycusis. Then the prognostic roles of the hub genes were analyzed and verified *in vivo*.

**Results:**

In the old mild hearing loss group (27.7 ± 3.4 months old), 27 down-regulated and 99 up-regulated genes were significantly differentially expressed compared with those in the young control group (3.5 ± 0.4 months old). In the old severe hearing loss group (30.6 ± 1.9 months old), 131 down-regulated and 89 up-regulated genes were significantly differentially expressed compared with those in the young control group. The results of the GO, GSEA, KEGG pathway, and immune infiltration analyses showed that the enrichment terms were mainly focused on immune response in mild presbycusis, and immune response and cell death in severe presbycusis. In the PPI network, autophagy-related genes *ATG5, ATG7* showed the highest node scores in mild presbycusis; whereas *MTOR, BECN1* showed the highest scores in severe presbycusis. In the GSE49543 data set, four genes (*Ywhag, Mapre2, Fgf1, Acss2*) were used to construct the prognostic model, and those four genes were significantly up-regulated in the rat model of presbycusis.

**Conclusion:**

Our study is the first to report the difference in autophagy factors and immune microenvironment among different degrees of hearing loss in presbycusis. Furthermore, we provide the prognostic gene expression signature for age-related hearing loss, intending to develop preventative therapies.

## Introduction

Age-related hearing loss, the most common sensory disease, is also known as presbycusis. Over one quarter of the people over 60 years of age are affected by hearing loss disabilities (1). Presbycusis is multifactorial, and involves both genetic and environmental factors (2). The disease is characterized by bilateral, symmetrical high-frequency sensorineural hearing loss that can range from mild to severe (3). Presbycusis not only reduces the ability of hearing sensitivity and speech understanding, but untreated hearing loss can also contribute to social isolation, depression, and cognitive decline (4, 5).

The pathology of human presbycusis is characterized by the loss of hair cells, degeneration of the spiral ganglion neurons, and cell death in the stria vascularis. Besides the peripheral portion, the central auditory pathways also change (4, 6). In this study, we have predominantly focused on the peripheral auditory system. The degeneration of the cochlear cells does not affect people uniformly; in fact, the degeneration process appears to be not uniform even within an individual (7). This is not only due to individual causal factors, but also due to the interaction of the different mechanistic pathways. Several genome-wide association studies have found that the role of a hereditary factor has been overestimated (3), and there may be different underlying mechanisms leading to the development of different degrees of presbycusis.

Further, there is a lack of effective and specific prognostic molecular markers for presbycusis, and this disease cannot be prevented or treated with drugs. With the rapid development of molecular biology, it is now possible to clarify the biochemical processes and molecular biology of presbycusis and develop drugs to either decline or improve presbycusis. Presbycusis is caused by an imbalance between pro-aging and anti-aging mechanisms. Inflammation, oxidative stress, apoptosis, accumulation of DNA damage, and autophagy can lead to aging (7). Many studies have used microarray technology to understand the molecular mechanisms in presbycusis. Screening for methylation map changes in peripheral blood samples of women with presbycusis as compared to controls, *P2RX2* (purinergic receptor P2 × 2), *KCNQ5* (potassium voltage-gated channel subfamily Q member 5), *ERBB3* (erb-b2 receptor tyrosine kinase 3), and *SOCS3* (suppressor of cytokine signaling 3) have been associated with the progression of age-related hearing loss (8). In CBA/CaJ mice, *Hspb1* [heat shock protein family B (small) member 1] shows differential expression between mild and severe presbycusis (9). However, no study has reported the difference in autophagy factors and immune microenvironment among different degrees of hearing loss in presbycusis, and previous studies mostly used single-chip analysis which can lead the false positive rate higher. Accordingly, further comprehensive analyses based on cross-validation are needed to identify more robust and reliable biomarkers for the progression and prognosis of presbycusis.

We downloaded microarray data sets (GSE6045 and GSE49543) from the Gene Expression Omnibus (GEO) database, and determined significant differentially expressed genes (DEGs) between old hearing loss groups and young normal control group. Gene Ontology (GO), Kyoto Encyclopedia of Genes and Genomes (KEGG) pathway, and protein-protein interaction (PPI) network analyses along with Gene set enrichment analysis (GSEA) were performed to provide more information about the molecular mechanisms of the DEGs. We also analyzed the difference in immune microenvironment between the old hearing loss groups and young normal control group in 22 types of immune cells. Moreover, prognostic molecular markers were validated for presbycusis. Thus, our study may provide evidence of molecular mechanisms underlying the pathology of presbycusis.

## Materials and Methods

### Data Source and Preprocessing

Microarray data sets were downloaded from the GEO database.^1^ Two gene expression datasets [GSE6045 (10), and GSE49543 (11)] based on the same GPL339 platform (Affymetrix Mouse Expression 430A Array) and annotated using the “GEOquery” R package (12) were acquired. The species selected was Mus musculus. GSE49543 was designated as the training set and GSE6045 as the testing set. Additionally, the GSE49543 data set was categorized into four groups: young control (young adults with good hearing), middle control (middle aged with good hearing), old mild hearing loss (old mice with mild hearing loss), and old severe hearing loss (old mice with severe hearing loss) groups. The GSE6045 dataset was classified into two groups: mild and severe hearing loss groups. The details and characteristics of mice in the two datasets are showed in Table 1.

**TABLE 1:** Details and characteristics of mice in the two datasets (GSE49543 and GSE6045).

	Platform	Microarray	Group	Sample size	Age (months)
GSE49543	GPL339	Affymetrix mouse expression 430A array	Young control	8 (excluding GSM1201567)	3.5 ± 0.4
			Middle control	17	12.3 ± 1.5
			Old mild hearing loss	9	27.7 ± 3.4
			Old severe hearing loss	6	30.6 ± 1.9
GSE6045	GPL339	Affymetrix mouse expression 430A array	Mild hearing loss	3	2
			Severe hearing loss	3	8

### Identification of the Differentially Expressed Genes

The “limma” R package (13) was applied to the microarray data to filter DEGs. In GSE49543, we compared the DEGs in the young control vs. the old mild hearing loss and old severe hearing loss groups. *P*-value < 0.05 and absolute value of logFC > mean {abs(logFC) + 2 × SD [abs(logFC)]}, where logFC is the log fold change value and SD is the standard deviation, were set as the cut-off criteria for determining the significant DEGs.

### Functional and Pathway Enrichment Analyses

Gene ontology analysis was performed to identify enrichment terms associated with the DEGs. The GO terms included molecular function (MF) and cellular component (CC). The mouse organism annotation package “org.Mm.eg.db” was used to retrieve the relevant annotations. GSEA (14) was conducted to demonstrate significant differences and identify the signaling pathways in the young control group and each old hearing loss group. GO analysis (*P*-value was set to <0.99), KEGG pathway analysis (*P*-value was set to <0.01), and GSEA (*P*-value was set to <0.25) were conducted in R (version 4.0.5) using the “clusterProfiler” package (15). The gene set permutations were performed 10,000 times.

### Protein-Protein Interaction Network Construction

A PPI network can help us clarify the pathogenesis and progression of diseases. The identified DEGs were mapped to the STRING database (version 10)^2^ to analyze the PPI of autophagy-related DEGs. The interactions with reliability scores more than 0.4 were selected for analysis. Cytoscape software (16) was applied to construct the PPI network and the plugin “cytoHubba” (17) was utilized to explore the PPI network. The top 20 percent of the hub genes were selected by “cytoHubba” based on the maximum correlation criterion algorithm. High ranked genes were represented by a redder color.

### Immune Infiltration Analysis

The marker genes for immune cell types were referred from a previous study (18). Infiltration levels of immune cells were analyzed using the single-sample GSEA method. Spearman’s correlation analysis was performed to show the association between different degrees of hearing loss and the infiltration of the immune cell types.

### Survival Analysis

We analyzed the DEGs between the old mice with hearing loss (*n* = 15) and middle-aged mice with good hearing (*n* = 17). The relationship between the clinicopathologic characteristics and gene expression were assessed using hazard ratio (HR). Univariate Cox regression analysis was performed to obtain the prognostic genes and multivariate Cox analysis was applied to construct a prognostic model. The Kaplan-Meier curves were used to study the effects of the prognostic model in the training set. As the previous study did not describe the survival time clearly (11), we set the survival time as 8 months for the middle-aged mice group with good hearing and 24 months for the old mice group with hearing loss.

### Animal Model and Treatment

Male Sprague Dawley rats (8 weeks old) were purchased from the Model Animal Research Center of Guangxi Medical University (Nanning, China). The rats were randomly divided into two groups: the control group and the presbycusis group (*n* = 4). All animals had no history of noise exposure and other drug use. ABR and DPOAE were performed before the experiment to determine their hearing normally. The rats in the presbycusis group were given 5% D-galactose (250 mg/kg) daily by intraperitoneal injection for 10 weeks; The control group was given a similar volume of saline daily by intraperitoneal injection for 10 weeks.

One day after the last administration, ABR and DPOAE were performed to ensure successful modeling. After recordings, animals were transcardially perfused and obtained cochlear tissue. The animal study was approved by the Institutional Ethics Committee for Animal Research of Guangxi Medical University. All procedures conformed to the Guide for the Care and Use of Laboratory Animals.

### Reverse Transcription-Polymerase Chain Reaction

Total cochlear mRNA was extracted using TRIzol reagent and reverse transcribed into cDNA. RT-qPCR was performed by StepOneP™ Real-Time PCR System (Thermo Fisher, Waltham, MA, United States) using the SYBERGreen kit of Takara company. The total volume of the reaction system is 20 μl [10 μl SYBERGreen (2×), 7 μl DEPCwater, 1 μl cDNA, 0.8 μl forward primer, 0.8 μl reverse primer, 0.4 μl Rox reference dye (50×)]. The reaction conditions were pre-incubated at 95°C for 30 s and one cycle, followed by denaturation at 95 °C for 10 s; and elongation at 60 °C for 30 sec, repeated for 45 cycles. Each sample was run in triplicate and averaged. The relative gene expression was calculated by the 2^–△^^△^ Ct method.

The primer sequence is as follows: *Fgf1* forward primer: 5′-AGCAGCAGGAATGCATTGAGG-3′, reverse primer: 5′-AACTGTCGATGGTGCGTTCAAG-3′. *Ywhag* forward primer: 5′- CAGCTGCTCCGAGACAACCTA-3′, reverse primer: 5′- AGGAACCATCCACGCTCA-3′. *Mapre2* forward primer: 5′- TGCCAAGACGCGTTAGCAG-3′, reverse primer: 5′- CAAGCAGCCAGGTGGTGAAG-3′. *Acss2* forward primer: 5′- GAACCACACACGTTTCGAGACC-3′, reverse primer: 5′- TCATCAATCCTGCCAGTGATCC-3′. β*-actin* forward primer: 5′- GCGCAAGTACTCTGTGTGGA-3′, reverse primer: 5′-GAAAGGGTGTAAAAC GCAGC-3′.

## Results

### Identification of the Differentially Expressed Genes in Presbycusis

**FIGURE 1:**
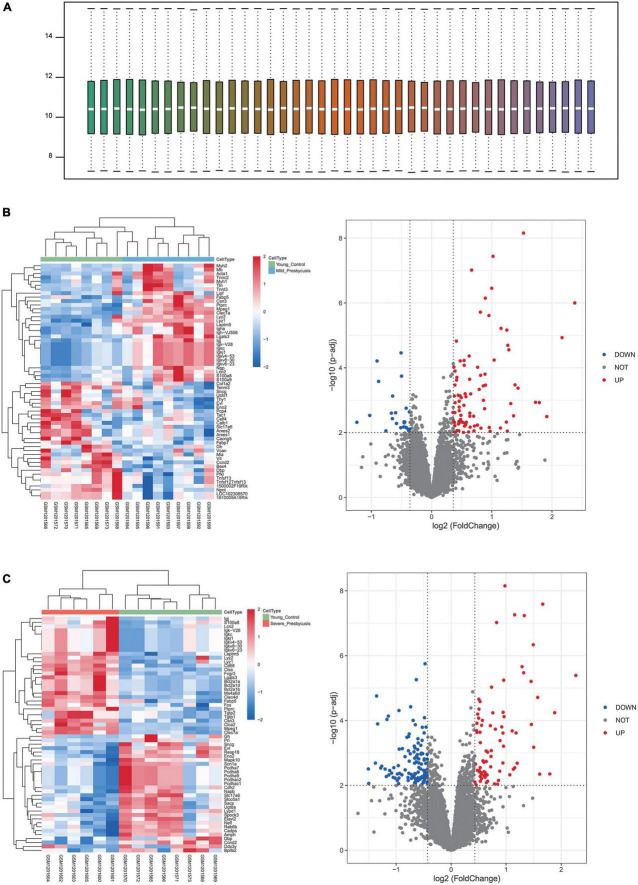
Identification of the differentially expressed genes (DEGs) in presbycusis in GSE49543. **(A)** Differentially expressed mRNAs data sets were standardized. **(B)** The heat map and volcano plot of the top 30 up-regulated (red dots) and top 30 down-regulated (blue dots) DEGs in mild presbycusis. **(C)** The heat map and volcano plot of the top 30 up-regulated (red dots) and top 30 down-regulated (blue dots) DEGs in severe presbycusis.

In GSE49543, each sample was standardized to the average gene expression (Figure 1A), the expression values for those genes were similar after normalized. GSM1201567 was excluded as it was not clustered with the other samples belonging to the control group in the heat maps. In the old-aged mild hearing loss group, a total of 126 DEGs were identified, which comprised 99 up-regulated (logFC > 0.36 and *P*-adj. <0.01) and 27 down-regulated genes (logFC < −0.36 and *P*-adj. <0.01). The heat map and volcano plot of the top 30 up-regulated and top 30 down-regulated genes are shown in Figure 1B (There were not enough down-regulated genes in the old-aged mild hearing loss group, so the heatmap shows all of them). In the old aged severe hearing loss group, a total of 220 DEGs were identified, which comprised 89 up-regulated (logFC > 0.43 and *P*-adj. <0.01) and 131 down-regulated genes (logFC < −0.43 and *P*-adj. <0.01). The heat map and volcano plot of the top 30 up-regulated and top 30 down-regulated genes are shown in Figure 1C. In the heatmap, each row represents one gene, and each column represents one sample. The color difference among samples is a result of gene expression differences. In the volcano plot, gray dots represent no significantly different expression genes, red dots represent significantly up-regulated genes, and green dots represent downregulated.

### Gene Ontology and Kyoto Encyclopedia of Genes and Genomes Pathway Analyses of the Differentially Expressed Genes

**FIGURE 2:**
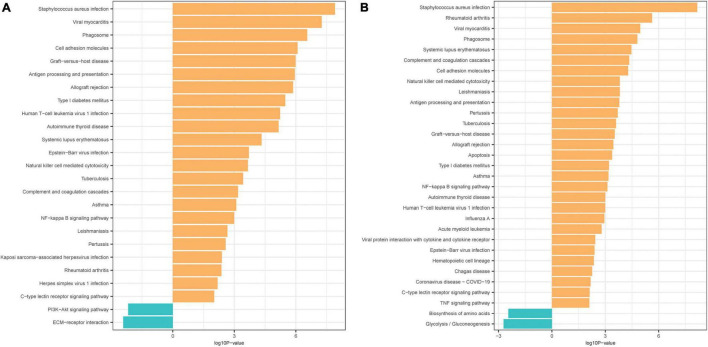
Kyoto Encyclopedia of Genes and Genomes (KEGG) pathway analyses of the DEGs. **(A)** KEGG pathway analysis of the DEGs in mild presbycusis. **(B)** KEGG pathway analysis of the DEGs in severe presbycusis. Each row represents one pathway, and column represents log10P-value.

In the mild hearing loss group, GO analysis showed that the DEGs were considerable enriched in the terms “immunoglobulin receptor binding,” “antigen binding,” “peptide antigen binding,” “acetylcholine receptor regulator activity,” “neurotransmitter receptor regulator activity,” and “acetylcholine receptor binding” in the MF category (Table 2), and in the terms “external side of plasma membrane,” “MHC protein complex,” “immunoglobulin complex,” “circulating,” “immunoglobulin complex,” “Golgi cisterna,” and “MHC class I protein complex” in the CC category (Table 2). KEGG pathway analysis indicated that the DEGs were mainly enriched in “*Staphylococcus aureus* infection,” “viral myocarditis,” “phagosome,” “antigen processing and presentation,” “graft-vs.-host disease,” and “cell adhesion molecules” (Figure 2A and Table 2). Meanwhile, in the severe hearing loss group, GO analysis showed that the DEGs were remarkably enriched in the terms “BH domain binding,” “Immunoglobulin receptor binding,” “receptor inhibitor activity,” “calcium ion binding,” “platelet-derived growth factor binding,” “acetylcholine receptor regulator activity” in the MF category (Table 2), and in the terms “external side of plasma membrane,” “MHC protein complex,” “myelin sheath,” “collagen trimer,” “focal adhesion,” and “phagocytic vesicle” in the CC category (Table 2). KEGG pathway analysis expressed that the DEGs were mainly enriched in the terms “*Staphylococcus aureus* infection,” “rheumatoid arthritis,” “viral myocarditis,” “phagosome,” “systemic lupus erythematosus,” and “cell adhesion molecules” (Figure 2B and Table 2).

**TABLE 2:** Gene ontology (GO) and Kyoto Encyclopedia of Genes and Genomes (KEGG) pathway enrichment analysis of differentially expressed genes (DEGs) in the most significant module.

Category	Mild presbycus				Severe presbycus			
	ID	Description	Count	*p*-adjust	ID	Description	Count	*p*-adjust
GOTERM_MF	GO:0034987	Immunoglobulin receptor binding	7	4.58E−08	GO:0051400	BH domain binding	4	2.16E−02
GOTERM_MF	GO:0003823	Antigen binding	9	2.78E−07	GO:0034987	Immunoglobulin receptor binding	4	4.78E−02
GOTERM_MF	GO:0042605	Peptide antigen binding	5	1.68E−04	GO:0030547	Receptor inhibitor activity	4	4.78E−02
GOTERM_MF	GO:0030548	Acetylcholine receptor regulator activity	4	1.68E−04	GO:0005509	Calcium ion binding	18	4.78E−02
GOTERM_MF	GO:0099602	Neurotransmitter receptor regulator activity	4	1.68E−04	GO:0048407	Platelet-derived growth factor binding	3	5.79E−02
GOTERM_MF	GO:0033130	Acetylcholine receptor binding	4	2.64E−04	GO:0030548	Acetylcholine receptor regulator activity	3	5.79E−02
GOTERM_CC	GO:0009897	External side of plasma membrane	19	1.27E−07	GO:0009897	External side of plasma membrane	17	6.39E−02
GOTERM_CC	GO:0042611	MHC protein complex	5	1.11E−04	GO:0042611	MHC protein complex	4	7.30E−02
GOTERM_CC	GO:0042571	Immunoglobulin complex, circulating	4	3.48E−04	GO:0043209	Myelin sheath	10	2.05E−01
GOTERM_CC	GO:0019814	Immunoglobulin complex	4	7.77E−04	GO:0005581	Collagen trimer	5	2.69E−01
GOTERM_CC	GO:0031985	Golgi cisterna	5	3.39E−03	GO:0005925	Focal adhesion	7	3.06E−01
GOTERM_CC	GO:0042612	MHC class I protein complex	3	3.39E−03	GO:0045335	Phagocytic vesicle	6	3.06E−01
KEGG_PATHWAY	mmu05150	*Staphylococcus aureus* infection	9	1.70E−06	mmu05150	*Staphylococcus aureus* infection	9	8.89E−07
KEGG_PATHWAY	mmu05416	Viral myocarditis	8	3.79E−06	mmu05323	Rheumatoid arthritis	7	1.54E−04
KEGG_PATHWAY	mmu04145	Phagosome	10	1.29E−05	mmu05416	Viral myocarditis	6	4.76E−04
KEGG_PATHWAY	mmu04612	Antigen processing and presentation	7	2.59E−05	mmu04145	Phagosome	8	5.21E−04
KEGG_PATHWAY	mmu05332	Graft-vs.-host disease	6	2.59E−05	mmu05322	Systemic lupus erythematosus	5	8.94E−04
KEGG_PATHWAY	mmu04514	Cell adhesion molecules	9	2.59E−05	mmu04514	Cell adhesion molecules	7	9.73E−04

*MF, molecular function; CC, cellular component; GO, gene ontology; KEGG, Kyoto Encyclopedia of Genes and Genomes.*

### Gene Set Enrichment Analysis of the Hearing Loss-Related Genes

**FIGURE 3:**
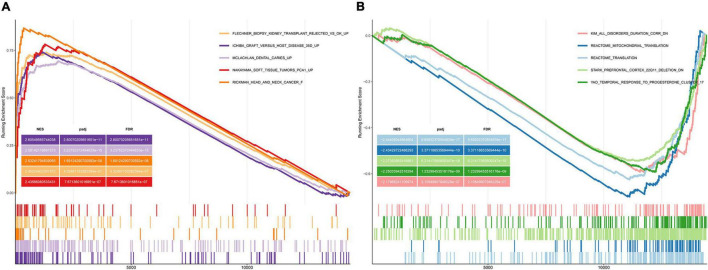
Gene Set Enrichment Analysis (GSEA) plots showing the most enriched gene sets in C2 collection in mild presbycusis. **(A)** The top five most significant up-regulated enriched gene sets and **(B)** the top five most significant down-regulated enriched gene sets in GSE49543.

**FIGURE 4:**
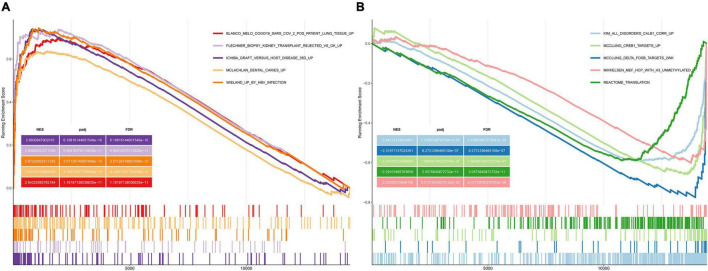
Gene Set Enrichment Analysis plots showing the most enriched gene sets in C2 collection in severe presbycusis. **(A)** The top five most significant up-regulated enriched gene sets and **(B)** the top five most significant down-regulated enriched gene sets in GSE49543.

We performed GSEA using the C2 (curated gene sets) collection. We identified biological pathways that were significantly altered in presbycusis groups compared with those in the young control group. The results of the gene expression profiles in GSE49543 are shown in Table 3. In the mild hearing loss group, the activated pathways were ichiba graft vs. host disease 35 d up, mclachlan dental caries up, rickman head and neck cancer f, flechner biopsy kidney transplant rejected vs. ok up, and nakayama soft tissue tumors pca1 up (Figure 3A); the suppressed pathways were: reactome translation, reactome mitochondrial translation, stark prefrontal cortex 22q11 deletion dn, yao temporal response to progesterone cluster 17, and kim all disorders duration corr dn (Figure 3B). In the severe hearing loss group, the activated enriched pathways were: ichiba graft vs. host disease 35 d up, flechner biopsy kidney transplant rejected vs. ok up, wieland up by hbv infection, mclachlan dental caries up, and blanco melo COVID-19 SARS-CoV-2 pos patient lung tissue up (Figure 4A); whereas, the suppressed pathways were: kim all disorders calb1 corr up, mcclung delta fosb targets 2 weeks, mcclung creb1 targets up, reactome translation, and mikkelsen mef hcp with h3 unmenthylated (Figure 4B). Together those shows that ichiba graft vs. host disease 35 d up pathway is a major pathogenic pathway of presbycusis, while reactome translation plays a protective role for inner ear. The abovementioned results indicated that the activated signaling pathways were mainly enriched in infection and immunity, whereas, the suppressed pathways were mainly enriched in the gene expression processes associated with presbycusis.

**TABLE 3:** Gene set enrichment analysis (GSEA) indicating statistically significant enrichment.

Mild presbycusis				Severe presbycusis			
Description	ES	NES	*p*-adjust	Description	ES	NES	*p*-adjust
REACTOME_TRANSLATION	−0.62573	−2.44408	9.66E−17	KIM_ALL_DISORDERS_CALB1_CORR_UP	−0.59203	−2.44131	2.54E−25
REACTOME_MITOCHONDRIAL_TRANSLATION	−0.70011	−2.4343	3.37E−10	MCCLUNG_DELTA_FOSB_TARGETS_2WK	−0.77549	−2.31971	6.27E−07
STARK_PREFRONTAL_CORTEX_22Q11_DELETION_DN	−0.54477	−2.27383	6.21E−18	MCCLUNG_CREB1_TARGETS_UP	−0.65934	−2.29165	1.97E−07
YAO_TEMPORAL_RESPONSE_TO_PROGESTERONE_ CLUSTER_17	−0.59278	−2.25034	1.23E−09	REACTOME_TRANSLATION	−0.59012	−2.29102	2.06E−11
KIM_ALL_DISORDERS_DURATION_CORR_DN	−0.59344	−2.17968	2.11E−07	MIKKELSEN_MEF_HCP_WITH_H3_UNMETHYLATED	−0.60184	−2.24356	5.07E−08
ICHIBA_GRAFT_VERSUS_HOST_DISEASE_35D_UP	0.741098	2.605496	2.80E−11	ICHIBA_GRAFT_VERSUS_HOST_DISEASE_35D_UP	0.737705	2.860085	6.20E−16
MCLACHLAN_DENTAL_CARIES_UP	0.693985	2.581421	3.24E−15	FLECHNER_BIOPSY_KIDNEY_TRANSPLANT_REJECTED_VS_ OK_UP	0.737343	2.698483	6.98E−11
RICKMAN_HEAD_AND_NECK_CANCER_F	0.867346	2.532418	1.69E−08	WIELAND_UP_BY_HBV_INFECTION	0.744022	2.672265	3.57E−10
FLECHNER_BIOPSY_KIDNEY_TRANSPLANT_REJECTED_VS_ OK_UP	0.739949	2.452244	4.33E−07	MCLACHLAN_DENTAL_CARIES_UP	0.636446	2.647871	4.20E−15
NAKAYAMA_SOFT_TISSUE_TUMORS_PCA1_UP	0.78259	2.435661	7.67E−07	BLANCO_MELO_COVID19_SARS_CoV_2_POS_PATIENT_LUNG_ TISSUE_UP	0.695495	2.64333	1.19E−11

*ES, enrichment score; NES: normalized enrichment score; E:10^.*

### Protein-Protein Interaction Network Construction and Evaluation

**FIGURE 5:**
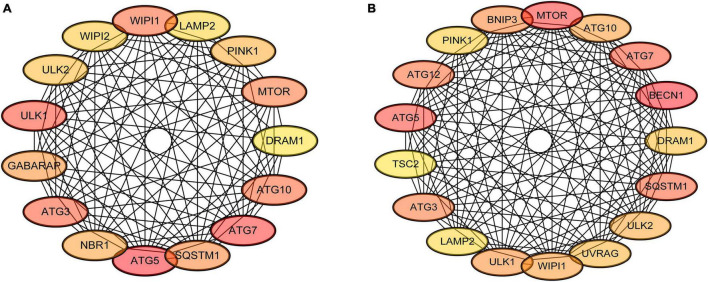
Protein-protein interaction (PPI) network construction and major autophagy-related genes. The cytoHubba plugin was used to analyze the top 20 percent of the hub genes with maximum correlation criterion in mild **(A)** and severe **(B)** presbycusis.

The interactions of the autophagy-related DEGs were analyzed using the STRING database, which comprised 72 DEGs in the mild hearing loss group and 88 DEGs in the severe hearing loss group. The top 20 percent of the autophagy-related hub genes were identified using the cytoHubba plugin (Figure 5). The darker the color, the higher node score is in the PPI network. Among these genes, ATG5, ATG7 showed the highest node scores in the mild hearing loss group, whereas MTOR, BECN1 showed the highest node scores in the severe hearing loss group. These results suggested that these genes may play an important role in the development or progression of presbycusis and different degree of hearing loss may induce autophagy by different mechanisms.

### The Relationship Between Hearing Loss Degree and Immune Infiltration

**FIGURE 6:**
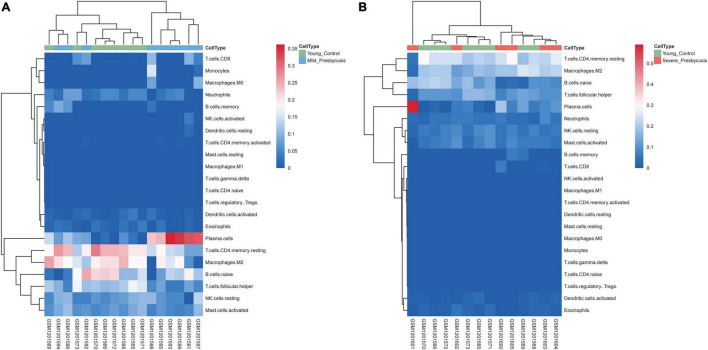
Landscape of immune infiltration in presbycusis. **(A)** The heat maps of different types of immune cells expression between mild presbycusis and young control group. **(B)** The heat maps of different types of immune cells expression between severe presbycusis and young control group.

**FIGURE 7:**
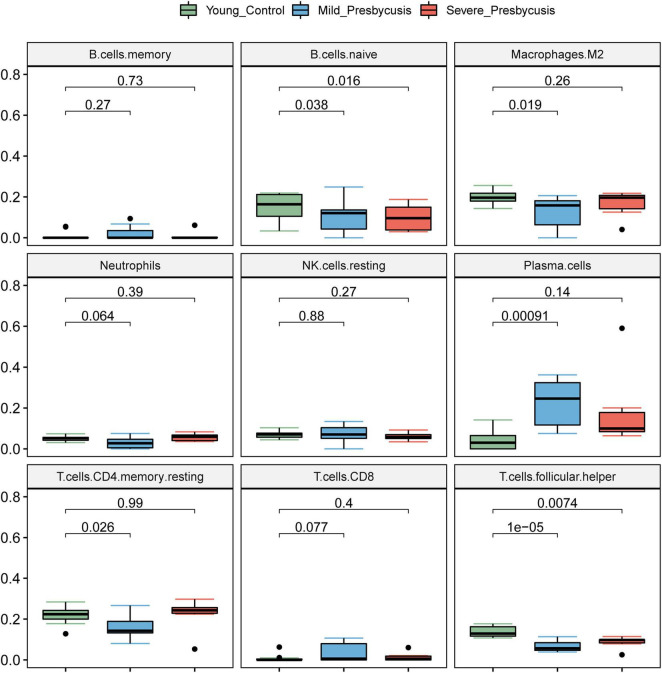
The relationship between hearing loss degree and immune infiltration. The differential expression of the immune cells between each presbycusis group and control group is shown.

We investigated the differences in the immune cell infiltration between the presbycusis cochlea tissues and control cochlea tissues. The differential expression of the immune cells between each presbycusis group and control group is shown in Figure 6. Each row represents one type of 22 immune cell, and each column represents one sample. Five immune cell subsets were differentially expressed: B cells naïve, plasma cells, CD4 memory resting T cells, follicular helper T cells, and M2 macrophages. The B naïve immune cells in the mild hearing loss group (*P* = 0.038) showed lower differential expression than that in the severe group (*P* = 0.016); the M2 macrophages in the mild group showed greater differential expression (*P* = 0.019) than that in the severe group (*P* = 0.26); the plasma cells (*P* < 0.001) in the mild group showed higher differential expression than that in the severe group (*P* = 0.14); the CD4 memory resting T cells in the mild group showed higher differential expression (*p* = 0.026) than that in the severe group (*P* = 0.99); and finally, the follicular helper T cells in the mild group showed higher differential expression (*p* < 0.001) than that in the severe group (*P* = 0.0074) (Figure 7).

### Prognostic Value of the Hearing Loss-Related Genes in Presbycusis

**FIGURE 8:**
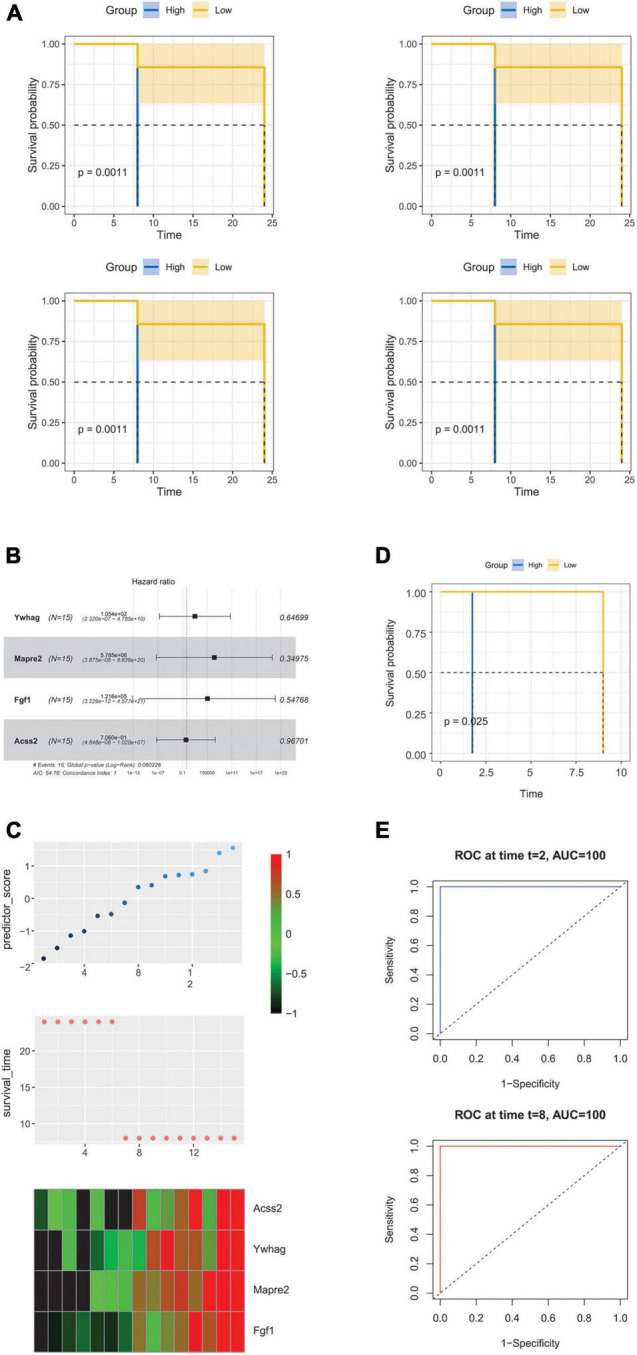
Prognostic value of the hearing loss-related genes in presbycusis. **(A)** KM curve of overall survival (OS) prognosis of four genes in GES49543. **(B)** Forest map of OS multivariate analysis of four genes in presbycusis. **(C)** Top: the risk score of prediction model in validation set; Middle: corresponding survival status of each sample; Bottom: the mRNA expression of four signature genes in each sample. **(D)** KM curve of overall survival (OS) prognosis in GES6045. **(E)** ROC curve and AUC of prognostic model in GSE6045.

Univariate Cox regression analysis was performed to identify the prognostic differentially expressed hearing loss related genes in GSE49543. Four genes were significantly correlated with the overall survival (OS) in presbycusis (Figure 8A). Considering the effect of the prognostic value, we further constructed a Cox regression hazard model: Risk score = −2.8 × (Ywhag + Mapre2 + Fgf1 + Acss2), where c-index is 0.745 (0.720–0.771). The risk scores for each gene were calculated using their expression levels and regression coefficients (Figure 8B). Higher risk scores indicated greater hearing loss risk for old people. The expression level of four signature genes (*n* = 15) from GSE49543 is shown in Figure 8C. A survival curve was drawn to validate the accuracy and repeatability of the four prognostic genes in GSE6045 (Figure 8D). We then used a time-dependent receiver operating characteristic (ROC) curve to confirm the predictive accuracy of the model, and the area under curve was 100% as the sample size was small (Figure 8E).

### The Expression Level of the Prognostic Genes *in vivo*

**FIGURE 9:**
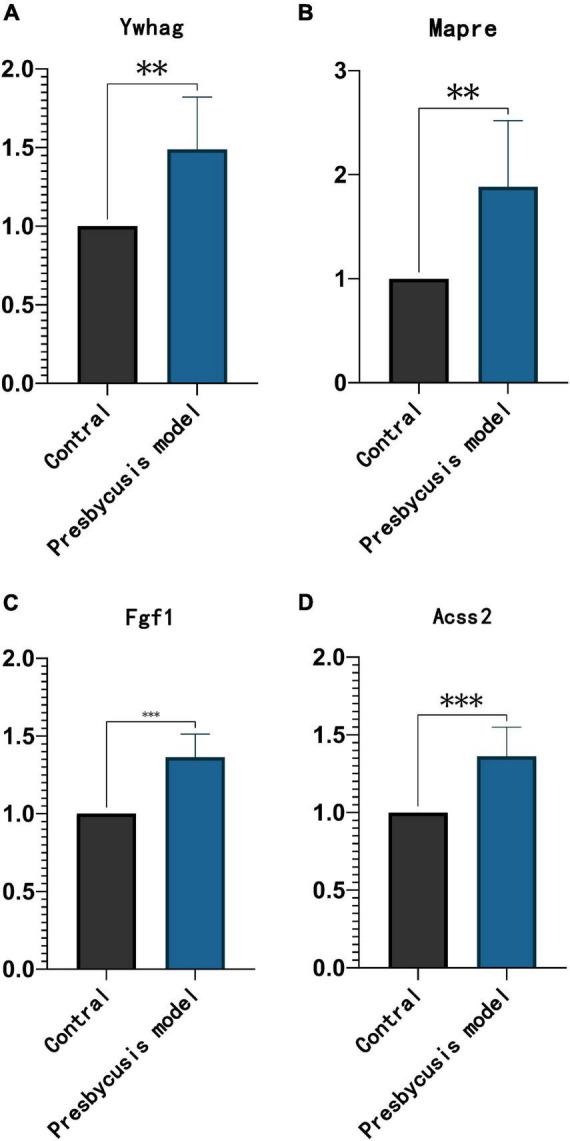
The expression trend of prognostic factors in the inner ear of D-galactose induced rats. The expression of Ywhag **(A)**, Mapre2 **(B)**, Fgf1 **(C)**, and Acss2 **(D)**. ^**^p < 0.01 and ^***^p < 0.001 compared with control.

Ten weeks post-intraperitoneal injection with D-galactose, rats are aging manifestations in hair, nerve reflex and hearing compared with contral rats. D-galactose-induced subacute aging model is most frequently used as an animal model for presbycusis. All of the four genes (*Ywhag, Mapre2, Fgf1*, and *Acss2*) were significantly up-regulated compared to the control group (Figure 9).

## Discussion

Presbycusis is a multifactorial disorder that is associated with genetic and environmental factors (3). While aging is inevitable, not everybody will suffer from presbycusis (7). Elucidating the mechanisms underlying the pathogenesis and progression of age-related hearing loss would aid in its prevention and therapy. With the technological advances of microarray and bioinformatic analyses, we can now identify the underlying molecular mechanisms of disease development and progression. In mammals, inner ear hair cells cannot easily regenerate after damage, and presbycusis animal models have been generated to study the pathogenesis and molecular basis.

Physiologically, autophagy maintains the internal environment of the body by degrading the cytoplasmic materials in lysosomes. When autophagy is dysregulated, it may cause adverse effects on the body (19, 20). Autophagy is enhanced in aging-related diseases (19–21), and increased autophagic stress occurs during premature age-related hearing loss in mice (22). In C57BL/6 mice, miR-34a/SIRT1 signaling activation can reduce age-related cochlear hair cell loss *via* regulated autophagy (23–25). Nevertheless, there are a few studies that have focused on autophagy in presbycusis. Thus, it is worth exploring whether there are differences between autophagy markers or their expression levels that are associated with different degrees of hearing loss in presbycusis.

Cancer, inflammation and aging are interconnected. Immune microenvironment determines the invasion capacity of the tumor cells, proliferation, and metastasis. Macrophages and T cells are the key components of the microenvironment, and immune checkpoint inhibitors have shown clinical benefits across a wide variety of tumor types (26, 27). Unlimited expression and production of inflammatory mediators are the key features of aging (28). As previously reported, aging has an impact on the Langerhans cells, dendritic cells, and natural killer cells. However, the role of the immune microenvironment in the pathogenesis of presbycusis remains unknown.

In this study, we performed an integrative analysis using two publicly available mouse mRNA microarray datasets (GSE6045 and GSE49543). On comparing the samples from the young control group, we observed that samples with different degrees of hearing loss (ranging from mild to severe) did not have the same DEGs. Three autophagy-related genes (Dram1, Fkbp1b, and Fos) were differentially expressed in the mild hearing loss group, whereas five autophagy-related genes (Dram1, Fkbp1b, Fos, Capn2, and Eef2) were differentially expressed in the severe hearing loss group. The difference in the expression of the biochemical markers indicated that the autophagy process was involved in both groups, although, with different degrees. Based on the PPI network analysis and hub gene identification, we found that the mild and severe hearing loss groups had different autophagy-related hub genes, suggesting that different degrees of hearing loss may occur in different autophagy pathways. It is likely activating autophagy activity is the main mechanism in mild presbycusis and stimulus-induced autophagy plays important roles in severe presbycusis. The difference between the two pathogenesis is worthy of further discussion.

Gene ontology analyses revealed that the main GO terms were enriched in immune response in the mild hearing loss group, and with immune response and cell death in the severe hearing loss group. KEGG enrichment analysis of the DEGs revealed that the most enriched pathways in our analysis were associated with immune response, inflammation, and cell death. This result is consistent with that in previous studies reporting that inflammation and autophagy were the key mechanisms associated with aging cochlea (22, 23, 29). By combining GSEA results, we found many DEGs that were important in infection and immunity. Results revealed that there were different signaling pathways and biological processes in the mild and severe hearing loss groups.

The inner ear is not an immune-privileged organ, which is why we attempted to reveal the differences in immune cell infiltration in presbycusis. A high expression of T follicular helper cells and B cells has been strongly associated with good prognosis in patients with human colorectal cancer (18). Our results also showed that the expression of T follicular helper cells and B cells was reduced in the presbycusis groups. M2 macrophages expression was reduced in the mild hearing loss group but remained unchanged in the severe hearing loss group. However, previous studies have reported macrophage invasion in the cochlear of presbycusis mice models (22). M1 macrophages are proinflammatory, whereas M2 macrophages are anti-inflammatory (30). So far, there is no research about the changes in macrophage subsets observed in presbycusis. Thus, the role of different types of immune cells in the pathogenesis of presbycusis still needs to be clarified.

Hearing loss occurs with aging in presbycusis, and we found four genes that were significantly associated with aging. Ywhag is a member of the 14-3-3 family, which mediats signal transduction by binding to phosphoserine-containing proteins. A study has reported the upregulation of Ywhag in age-related hearing loss (31). Hypoxia can activate p53 through the inactivation of MDMX by the ATR-Chk1-MDMX-14-3-3γ pathway (32). Mapre2, a microtubule-associated protein RP/EB family member 2, impacts the microtubule formation in the developing organ of Corti. Normal fluid spaces in cochlea are necessary for proper hearing (33). Fgf1 is involved in a variety of biological processes, and belongs to the fibroblast growth factor (FGF) family. It is involved in several cell activities, such as embryonic development, morphogenesis, cell growth, tissue repair, tumor growth, and invasion. FGFs possibly regulate the activity of signaling axonal growth or intrinsic neurons in the cochlear nucleus of adult mice following acoustic overstimulation (34). Acss2 is a cytosolic enzyme, which can catalyze the activation of acetate for its use in lipid synthesis and energy generation. Acss2 produces acetyl-CoA from acetate in an ATP-dependent reaction, and ATP levels are declined in D-galactose-induced aging mouse (35). However, there is no literature suggesting the relationship between Acss2 and presbycusis. Those four genes vertified increased in presbycusis *in vivo* experiment. Our study indicates that this may be a new prognostic factor of presbycusis. Further analyses were necessary to analyze through which pathway are those genes involved in regulation of presbycusis.

There are two main limitations of the present study. First, in order to clarify the mechanism of presbycusis, more clinical information needs to be considered, such as gender, age of onset, whether there are deafness susceptibility genes, etc. The prognostic factors of presbycusis, such as the degree of hearing loss and sex were not considered, and such a lack of consideration may have led to some biological information being overlooked. Second, the datasets based on the same sequencing company and sequencing platform is limit, thus the sample size in our study was not large enough. Further, *in vivo* and *in vitro* experiments are necessary to verify the association of these genes and pathways with presbycusis. Therefore, we aim to further increase the sample size and more clinical information to explore the pathogenesis of presbycusis. A series of molecular experiments may provide strong evidence for the possible phenotype and pathway regulation of these predicted genes recently.

To the best of our knowledge, this is the first study to report the difference in autophagy factors and immune microenvironment in different degrees of hearing loss in presbycusis. We used two microarray datasets to increase the reliability of our analysis. All data were downloaded from reliable GEO datasets. Furthermore, GO and KEGG analyses and GSEA were further carried out to analyze the main biological functions that were modulated by the DEGs. These data suggest that multiple biomarkers and pathways of presbycusis, in addition to different degrees of hearing loss may be caused by different molecular mechanisms in presbycusis. Thus, these results provide potential targets that can be employed to reduce or improve presbycusis progression.

## Conclusion

In this study, we combined two datasets to explore the molecular mechanisms associated with different degrees of hearing loss in presbycusis. Our results revealed DEGs, molecular pathways, immune cell infiltration and prognostic molecular markers affecting hearing impairment in presbycusis. This study provided a molecular basis of mildly and severely impaired auditory functions, with the aim of developing preventative therapies. However, more molecular experiments, both *in vivo* and *in vitro* are needed to validate the findings of the current study.

## Data Availability Statement

The datasets presented in this study can be found in online repositories. The names of the repository/repositories and accession number(s) can be found below: https://www.ncbi.nlm.nih.gov/geo/query/acc.cgi?acc=GSE49543 and https://www.ncbi.nlm.nih.gov/geo/query/acc.cgi?acc=GSE6045.

## Ethics Statement

The animal study was reviewed and approved by the Institutional Ethics Committee for Animal Research of Guangxi Medical University.

## Author Contributions

SY conceived and designed the study. NL and ZH performed data analysis. CQ contributed analysis tools. LP wrote the manuscript. All authors read and approved the final manuscript.

## Conflict of Interest

The authors declare that the research was conducted in the absence of any commercial or financial relationships that could be construed as a potential conflict of interest.

## Publisher’s Note

All claims expressed in this article are solely those of the authors and do not necessarily represent those of their affiliated organizations, or those of the publisher, the editors and the reviewers. Any product that may be evaluated in this article, or claim that may be made by its manufacturer, is not guaranteed or endorsed by the publisher.

## Footnotes

1
http://www.ncbi.nlm.nih.gov/geo

2
http://string-db.org

## References

[1] World Health Organization [WHO]. *Deafness and Hearing Loss.* (2020). Available online at: https://www.who.int/news-room/fact-sheets/detail/deafness-and-hearing-loss (accessed April 1, 2021).

[2] Ingham NJ Rook V Di Domenico F James E Lewis MA Girotto G Functional analysis of candidate genes from genome-wide association studies of hearing. *Hear Res.* (2020) 387:107879. 10.1016/j.heares.2019.107879 31927188 PMC6996162

[3] Bowl MR Dawson SJ. Age-related hearing loss. *Cold Spring Harb Perspect Med.* (2019) 9:a033217. 10.1101/cshperspect.a033217 PMC6671929 30291149

[4] Gates G Mills J. Presbycusis. *Lancet.* (2005) 366:1111–20. 10.1016/s0140-6736(05)67423-5 16182900

[5] Rutherford B Brewster K Golub J Kim A Roose S. Sensation and psychiatry: linking age-related hearing loss to late-life depression and cognitive decline. *Am J Psychiatry.* (2018) 175:215–24. 10.1176/appi.ajp.2017.17040423 29202654 PMC5849471

[6] Pal I Paltati C Kaur C Shubhi S Kumar P Jacob T Morphological and neurochemical changes in GABAergic neurons of the aging human inferior colliculus. *Hear Res.* (2019) 377:318–29. 10.1016/j.heares.2019.02.005 30878270

[7] Wang J Puel J-L. Presbycusis: an update on cochlear mechanisms and therapies. *J Clin Med.* (2020) 9:218. 10.3390/jcm9010218 31947524 PMC7019248

[8] Bouzid A Smeti I Dhouib L Roche M Achour I Khalfallah A Down-expression of P2RX2, KCNQ5, ERBB3 and SOCS3 through DNA hypermethylation in elderly women with presbycusis. *Biomarkers.* (2018) 23:347–56. 10.1080/1354750x.2018.1427795 29325454

[9] Tadros SF D’Souza M Zhu X Frisina RD. Gene expression changes for antioxidants pathways in the mouse cochlea: relations to age-related hearing deficits. *PLoS One.* (2014) 9:e90279. 10.1371/journal.pone.0090279 24587312 PMC3938674

[10] Someya S Yamasoba T Prolla TA Tanokura M. Genes encoding mitochondrial respiratory chain components are profoundly down-regulated with aging in the cochlea of DBA/2J mice. *Brain Res.* (2007) 1182:26–33. 10.1016/j.brainres.2007.08.090 17964557

[11] D’Souza M Zhu X Frisina RD. Novel approach to select genes from RMA normalized microarray data using functional hearing tests in aging mice. *J Neurosci Methods.* (2008) 171:279–87. 10.1016/j.jneumeth.2008.02.022 18455804 PMC2440495

[12] Davis S Meltzer PJB. GEOquery: a bridge between the Gene Expression Omnibus (GEO) and BioConductor. *Bioinformatics.* (2007) 23:1846–7. 10.1093/bioinformatics/btm254 17496320

[13] Smyth GK. Limma: linear models for microarray data. In: Gentleman R Carey VJ Huber W Irizarry RA Dudoit S *Bioinformatics and Computational Biology Solutions Using R and Bioconductor. Statistics for Biology and Health.* New York, NY: Springer (2005).

[14] Subramanian A Tamayo P Mootha VK Mukherjee S Ebert BL Gillette MA Gene set enrichment analysis: a knowledge-based approach for interpreting genome-wide expression profiles. *Proc Natl Acad Sci USA.* (2005) 102:15545–50. 10.1073/pnas.0506580102 16199517 PMC1239896

[15] Yu G Wang LG Han Y He QY. clusterProfiler: an R package for comparing biological themes among gene clusters. *OMICS.* (2012) 16:284–7. 10.1089/omi.2011.0118 22455463 PMC3339379

[16] Shannon P Markiel A Ozier O Baliga NS Wang JT Ramage D Cytoscape: a software environment for integrated models of biomolecular interaction networks. *Genome Res.* (2003) 13:2498–504. 10.1101/gr.1239303 14597658 PMC403769

[17] Chin CH Chen SH Wu HH Ho CW Ko MT Lin CY. cytoHubba: identifying hub objects and sub-networks from complex interactome. *BMC Syst Biol.* (2014) 8:S11. 10.1186/1752-0509-8-s4-s11 25521941 PMC4290687

[18] Bindea G Mlecnik B Tosolini M Kirilovsky A Waldner M Obenauf A Spatiotemporal dynamics of intratumoral immune cells reveal the immune landscape in human cancer. *Immunity.* (2013) 39:782–95. 10.1016/j.immuni.2013.10.003 24138885

[19] Mizushima N Levine B. Autophagy in human diseases. *N Engl J Med.* (2020) 383:1564–76. 10.1056/NEJMra2022774 33053285

[20] Stagni V Ferri A Cirotti C Barilà D. ATM kinase-dependent regulation of autophagy: a key player in senescence?. *Front Cell Dev Biol.* (2020) 8:599048. 10.3389/fcell.2020.599048 33490066 PMC7817534

[21] Chen M Hong C Yue T Li H Duan R Hu W Inhibition of miR-331-3p and miR-9-5p ameliorates Alzheimer’s disease by enhancing autophagy. *Theranostics.* (2021) 11:2395–409. 10.7150/thno.47408 33500732 PMC7797673

[22] Menardo J Tang Y Ladrech S Lenoir M Casas F Michel C Oxidative stress, inflammation, and autophagic stress as the key mechanisms of premature age-related hearing loss in SAMP8 mouse Cochlea. *Antioxid Redox Signal.* (2012) 16:263–74. 10.1089/ars.2011.4037 21923553

[23] Pang J Xiong H Lin P Lai L Yang H Liu Y Activation of miR-34a impairs autophagic flux and promotes cochlear cell death via repressing ATG9A: implications for age-related hearing loss. *Cell Death Dis.* (2017) 8:e3079. 10.1038/cddis.2017.462 28981097 PMC5680584

[24] Pang J Xiong H Ou Y Yang H Xu Y Chen S SIRT1 protects cochlear hair cell and delays age-related hearing loss via autophagy. *Neurobiol Aging.* (2019) 80:127–37. 10.1016/j.neurobiolaging.2019.04.003 31170533

[25] Xiong H Chen S Lai L Yang H Xu Y Pang J Modulation of miR-34a/SIRT1 signaling protects cochlear hair cells against oxidative stress and delays age-related hearing loss through coordinated regulation of mitophagy and mitochondrial biogenesis. *Neurobiol Aging.* (2019) 79:30–42. 10.1016/j.neurobiolaging.2019.03.013 31026620

[26] Chevrier S Levine J Zanotelli V Silina K Schulz D Bacac M An immune atlas of clear cell renal cell carcinoma. *Cell.* (2017) 169:736–49.e18. 10.1016/j.cell.2017.04.016 28475899 PMC5422211

[27] Kumagai S Togashi Y Kamada T Sugiyama E Nishinakamura H Takeuchi Y The PD-1 expression balance between effector and regulatory T cells predicts the clinical efficacy of PD-1 blockade therapies. *Nat Immunol.* (2020) 21:1346–58. 10.1038/s41590-020-0769-3 32868929

[28] Bottazzi B Riboli E Mantovani A. Aging, inflammation and cancer. *Semin Immunol.* (2018) 40:74–82. 10.1016/j.smim.2018.10.011 30409538

[29] Watson N Ding B Zhu X Frisina RJ. Chronic inflammation - inflammaging - in the ageing cochlea: a novel target for future presbycusis therapy. *Ageing Res Rev.* (2017) 40:142–8. 10.1016/j.arr.2017.10.002 29017893 PMC5675822

[30] Xia Y Rao L Yao H Wang Z Ning P Chen XJ. Engineering macrophages for cancer immunotherapy and drug delivery. *Adv Mater.* (2020) 32:e2002054. 10.1002/adma.202002054 32856350

[31] Yolande T Frisina RD D’Souza M. A novel high-throughput analysis approach: immune response-related genes are upregulated in age-related hearing loss. *Open Access Bioinform.* (2011) 3:107–22. 10.2147/oab.s13312

[32] Lee JH Jin Y He G Zeng SX Wang YV Wahl GM Hypoxia activates tumor suppressor p53 by inducing ATR-Chk1 kinase cascade-mediated phosphorylation and consequent 14-3-3γ inactivation of MDMX protein. *J Biol Chem.* (2012) 287:20898–903. 10.1074/jbc.M111.336875 22556425 PMC3375513

[33] Szarama K Stepanyan R Petralia R Gavara N Frolenkov G Kelley M Fibroblast growth factor receptor 3 regulates microtubule formation and cell surface mechanical properties in the developing organ of Corti. *Bioarchitecture.* (2012) 2:214–9. 10.4161/bioa.22332 23267415 PMC3527316

[34] Smith L Gross J Morest DJHR. Fibroblast growth factors (FGFs) in the cochlear nucleus of the adult mouse following acoustic overstimulation. *Hear Res.* (2002) 169:1–12. 10.1016/s0378-5955(02)00461-6 12121735

[35] Du Z Han S Qu T Guo B Yu S Wei W Age-related insult of cochlear ribbon synapses: an early-onset contributor to D-galactose-induced aging in mice. *Neurochem Int.* (2020) 133:104649. 10.1016/j.neuint.2019.104649 31870891

